# Impact of health conditions on daily functioning in Kenyan populations: A scoping review

**DOI:** 10.4102/ajod.v14i0.1456

**Published:** 2025-04-24

**Authors:** Naomi W. Kingau, Quinette A. Louw, Maria Y. Charumbira

**Affiliations:** 1 Department of Orthopedics and Rehabilitation, Moi University, Eldoret, Kenya; 2 Department of Physiotherapy, Faculty of Community and Health Sciences (CHS), Stellenbosch University, Cape Town, South Africa

**Keywords:** activity limitation, functional impairment, functional loss, disability, participation restriction

## Abstract

**Background:**

Kenya faces significant challenges in addressing the impact of various health conditions. Understanding the functioning problems associated with these conditions is crucial for informing targeted interventions and improving overall healthcare outcomes.

**Objectives:**

This study aimed to determine the prevalence and types of functioning problems associated with health conditions contributing most to Years Lived with Disability in the adult Kenyan population and to identify the International Classification of Functioning, Disability, and Health (ICF) domains and categories most affected.

**Method:**

A scoping review was conducted. Searches were performed across multiple databases using relevant keywords and inclusion criteria. Studies published between January 2006 and December 2023 were eligible. Data were extracted from 39 eligible studies using a web-based software application (*Rehab4all*).

**Results:**

Major depressive disorder, human immunodeficiency virus, low back pain and fractures were identified as the leading conditions contributing to functioning problems in Kenya. The most prevalent problems included walking difficulties, paraesthesia, various forms of pain and depression. The most affected ICF domains were mobility (d4), sensory function and pain (b2) and mental (b1).

**Conclusion:**

The comprehensive description of functioning problems associated with priority health conditions in Kenya can be used to develop targeted interventions to improve health outcomes across affected domains.

**Contribution:**

This research enhances comprehension of disability burden guiding intervention development and policy formulation for improved rehabilitation and offers a platform for further evidence-based strategies to tackle the country’s complex health challenges.

## Introduction

The capacity to engage in daily tasks and actively participate in personal and community activities serves as a crucial indicator of overall population health and well-being (Stucki et al. 2018). Long-term, short-term, or occasional impairment in functioning (ability to perform activities or tasks in their daily life) resulting from non-communicable diseases (NCDs), trauma, ageing and other conditions can significantly impact individuals’ ability to carry out these activities (Gyasi, Aboderin & Asiki 2022). Reduced functioning has a negative influence on individuals’ perceptions of their health status (Duntava, Borisova & Mäkinen 2021). Consequently, challenges in functioning exacerbate the impact on the perception of health and overall quality of life, particularly for individuals already dealing with morbidity associated with chronic conditions (Duntava et al. 2021).

Morbidity at a national level is often described using Global Burden of Disease (GBD) metrics such as Years Lived with Disability (YLDs) (Murray & Scola 2010). The YLDs reflect the total years lived with a chronic condition disease (Global Health Matrics 2018). Years Lived with Disability is on the rise at a faster rate in low- and middle-income countries (LMICs), compared to high-income countries (HICs) (Jesus, Landry & Hoenig 2019). According to GBD Compare (a data visualization tool by the Institute for Health Metrics and Evaluation [IHME]), the 2019 GBD in Kenya included depression, lower back pain (LBP), hearing problems, gynaecological conditions, headaches, human immunodeficiency virus (HIV), anxiety, iron deficiency, musculoskeletal conditions and oral health. Similarly, Kenyan Population Report of 2020 shows that about 1 million people are living with a disability, constituting 1.95% of the Kenyan population (Kenya National Bureau of Statistics 2022). Therefore, health systems in LMICs must adapt to the changing epidemiological landscape characterised by elevated levels of morbidity and problems related to functioning (Madden et al. 2012).

To equip LMICs, the World Health Organization (WHO) has initiated efforts to strengthen rehabilitation as a primary health strategy aimed at addressing functioning problems (Stucki et al. 2018). These WHO initiatives include a set of evidence-based rehabilitation interventions that should be given priority for integration into health systems (Rauch, Negrini & Cieza 2019). In addition, the WHO has introduced the Rehabilitation Competency Framework (RCF) to ensure that the rehabilitation workforce possesses the necessary skills and capabilities to address the rehabilitation needs of their respective populations (Mills et al. 2021). Recognising the diverse healthcare experiences and rehabilitation requirements across different countries, it is important to develop country-specific functioning profiles to effectively plan and integrate rehabilitation services into local health systems. Prioritisation is essential because of the limitations of resources, making data crucial in the decision-making process.

Integrating rehabilitation into the health systems of LMICs poses significant challenges. Using Kenya as an illustrative case, despite being classified as a middle-income country, it grapples with a substantial disease impact, including both communicable and NCDs (Ministry of Health 2021). The health system in Kenya faces constraints and fragmentation, with rehabilitation services being available mainly in national, county, and some sub-county hospitals (Ministry of Health 2021). A 2019 survey conducted by the Ministry of Health on Rehabilitative Services revealed that Kenya has less than 1500 rehabilitative professionals spread across national, county and some sub-county hospitals, despite the population reaching approximately 54 million (WHO 2019). This shortage significantly impacts the quality and accessibility of rehabilitative services, leading to unmet needs.

The private health sector provides healthcare services to a very small percentage of the population in Kenya (World Bank 2022). The majority of the Kenyans living in poverty receive healthcare from the public sector. which uses 9.5% of the allocated national healthcare budget (World Bank 2022). The limited public sector resources have to fund many competing health needs considering the high burden of disease (Charumbira et al. 2022a). Kenya’s prevalence of multimorbidity is at 28.7% of the 54 million and is associated with varying degrees of disability and functioning problems (Kenya National Bureau of Statistics 2022; Mohamed et al. 2019). Disability and issues with functioning often go unaddressed, particularly within primary healthcare settings where there is either a limited or non-existent rehabilitation workforce (Ministry of Health 2020). Thus, integrating rehabilitation services will require strong advocacy supported by appropriate national-level data on functioning needs to inform cost-effective and contextually relevant service planning. Kenya is currently reforming towards a Universal Health Coverage (Ministry of Health 2020). The rehabilitative and assistive technology strategic plan 2022–2026 within Universal Health Coverage promises that disability and rehabilitation services will be fully integrated into national, county and sub-county hospitals with a view to enhancing access to care (Ministry of Health 2020).

The International Classification of Functioning, Disability and Health (ICF) framework provides standard terminology for describing functioning (WHO 2013). This framework serves as a tool to identify functioning problems, including body impairments, activity limitations or participation restrictions that arise from an individual with a health condition interacting with contextual factors such as environmental and personal factors (Charumbira et al. 2022a; WHO 2013). The focus is removed from the health condition an individual presents with, to what they have difficulty doing – for example, being more concerned with whether a person has difficulty walking rather than whether the person has diabetes. The ICF framework is aetiologically neutral and serves to classify data on functioning across health conditions (Charumbira et al. 2022b; WHO 2013).

Comprehensive and comparable data on functioning in LMICs, especially in Kenya, is limited. Existing literature utilising the minimal generic ICF set (Cieza et al. 2014) is predominantly derived from HICs. This potentially lacks generalisability to Kenya’s context with constrained access to quality healthcare and unique profiles of conditions leading to disabilities (Jesus et al. 2019). Instruments such as the Washington Group Short Set on Functioning may not cover all critical ICF domains, potentially resulting in underreporting of functioning problems (Mitra et al. 2022). The World Bank Model Disability Survey, although more comprehensive, has not been implemented in Kenya. Current country-level planning often relies on GBD studies (WHO 2023), revealing a gap in detailed descriptions of functioning problems crucial for planning rehabilitation services.

Currently, there is a lack of clinical data estimates on the functioning problems of adult populations in Kenya. Conducting a comprehensive mapping of this data, compared across various health conditions, would offer valuable country-level insights, aiding in the planning of rehabilitation services (Gutenbrunner et al. 2018). Therefore, objectives of the review were to: (1) determine the prevalence and types of functioning problems (impairments, activity limitations and participation restrictions) associated with health conditions contributing most to YLDs in the adult Kenyan population and (2) identify the ICF domains and categories most affected.

This process involved identifying the top 10 conditions contributing the most to YLD in Kenya using the GBD Compare tool and for which evidence-based rehabilitation interventions exist (Cieza et al. 2019). Thus, existing peer-reviewed studies reporting on the prevalence of functioning problems in Kenyan adults diagnosed with any of the top 10 health conditions were searched. The most prevalent functioning problems reported in the studies were subsequently mapped to the ICF framework.

## Methods

A scoping review was conducted using an adapted framework by Arksey & O’Malley (2005), with updates from Hasanoff et al. (2024), to examine evidence on functioning problems related to priority conditions in Kenya. The ICF framework guided the review for standardised analysis of impairments, activity limitations and participation restrictions associated with common conditions. The review followed the Preferred Reporting Items for Systematic Reviews and Meta-Analyses for Scoping Reviews (PRISMA-ScR) guidelines (Tricco et al. 2018) for reporting scoping reviews.

### Review findings

This provides a guideline for the entire review process, to clearly define the the extent of the literature scoping process. In line with the purpose of scoping reviews, our approach was broad, with emphasis on studies that reported on functioning problems in Kenya.

### The review questions

1. What is the prevalence and type of functioning problems associated with health conditions contributing most to YLDs in Kenya?
2. Which are the ICF domains and categories affected by the most prevalent functioning problems in adult Kenya?

Eligibility of the research questions was informed by the Population, Exposure, Context, Outcome design (PECOd) framework (Peters et al. 2020). This included the following:

- Population (P) of patients 18+ years
- Exposure to at least one of the conditions contributing to the greatest YLD, as indicated by GBD 2019 data
- Context of Kenya
- Outcomes (O) was the functioning problems investigated
- All peer-reviewed study designs (D). (Charumbira et al. 2022a).

## Identifying relevant studies

### Search strategy

A comprehensive search was conducted between 01 January 2006 and 31 December 2023, across multiple electronic databases, reference lists and key journals, following the approach of a similar study in South Africa (Charumbira et al. 2022b). Databases searched included PubMed/MEDLINE, Scopus, Web of Science, EbscoHost (CINAHL and Africawide Information), SpringerLink, Cochrane Library, Science Direct, Embase and Sabinet. The search strategy targeted titles and abstracts. Key search terms are as follows: ‘activity limitation’, ‘functional impairment’, ‘functional loss’, ‘disability’ and ‘participation restriction’ used in different combinations alongside the search terms for the specified health conditions. Additional terms were added after analysing titles and abstracts, and the reference lists of eligible studies were manually reviewed to ensure thorough coverage.

### Eligibility criteria

#### Inclusion criteria

- Literature reported in English; the most common languages for scholarly communication in Kenya.
- Availability of full texts.
- Reporting information regarding functioning problems associated with conditions contributing most to YLDs in Kenya.

#### Exclusion criteria

- The study excluded impairments that were not indicated for rehabilitation such as health-related quality of life studies (focus on the individual’s values and expectations following disease or injury rather than functioning problems in terms of impairments, activity limitations and participation restrictions [Charumbira et al. 2022b]).
- Studies lacking prevalence information on functioning problems were excluded.
- The study did not consider grey literature^1^ (exclusively sought peer-reviewed published data and).

All database search results were transferred to Rayyan reference management software. Deduplication of all retrieved articles was performed in Rayyan prior to the initial phase of screening by title and abstract (Charumbira et al. 2022b).

### Screening process

The study selection process involved a two-step approach:

1. A single reviewer (N.W.) with the assistance of the librarian evaluated all titles and abstracts of retrieved articles using predetermined criteria to assess their potential eligibility. A second reviewer (M.C.) was consulted for additional input.
2. The initial reviewer (N.W.) conducted a more in-depth assessment by reviewing the full texts of eligible articles to ensure that they contained the necessary information. In instances of disagreement, decisions were reached through discussion and consensus.

### Data charting process and data items

Data were extracted from all eligible studies through the use of a web-based software application (*Rehab4all*). *Rehab4all* is a customised application that automates the visualisation of functioning problems, facilitating comparisons within and across conditions at the country level. The application enables the electronic entry of information extracted from eligible peer-reviewed publications (secondary data) on functioning problems. The application incorporates automated data synthesis functions, offering real-time outputs on the most prevalent functioning problems within and across health conditions. This feature permits regular updates as more peer-reviewed studies become available. In addition, the application automates the mapping of functioning problems to the ICF framework, ensuring consistent terminology for describing function (Charumbira et al. 2022a)

Data extracted included article title, first author, publication year, study design (quantitative studies), age (average or a range), sample population, location (rural, urban, semi-urban), care level (specialised hospital, hospital, primary healthcare, community, rehabilitation facility), gender, health condition (using the International Classification of Diseases 11th revision (ICD-11) and GBD Institute for Health Metrics), multimorbidity, and outcome measures for assessing function, types, and prevalence of functioning problems. Synonyms representing the same functioning problem were consolidated by selecting the most common term. For instance, ‘tingling’, ‘pins and needles’ and ‘numbness’ were represented as ‘paraesthesia’.

Data on the most common functioning problems at distinct recall periods (point, annual or lifetime) were extracted. In longitudinal studies reporting both baseline and post-intervention prevalence, the baseline was defined as the point at which the patient potentially initiates rehabilitation (Vollmar, Ostermann & Redaèlli 2015). This approach was even applied to conditions where rehabilitation follows medical interventions, to provide the worst-case scenario for strategic planning. No author contact was necessary to clarify or complete the data.

### Quality assessment

A methodological appraisal to assess the quality or risk of bias in the included studies was not conducted, in accordance with the scoping review methodological framework outlined by Levac, Colquhoun and O’Brien (2010) and supported by Hasanoff et al. (2024).

### Data analysis

Data analysis was facilitated by *Rehab4all* application. This application analysed health conditions, the level of care, the health setting and the study design. Furthermore, it analysed the prevalence of functioning problems and the number of articles related to them at the country level. The *Rehab4all* application presented the data through bar graphs and tables, while also mapping functioning problems onto the ICF domains in a spider web format.

### Type of functioning problems

The types of functioning problems presented in the included articles were classified using the ICF framework, with assistance from the *Rehab4all* application. In the ICF framework, first-level classifications are represented by letters: ‘b’ for body functions (eight domains), ‘d’ for activities and participation (nine domains), ‘e’ environmental factors (five domains) and ‘s’ for body structures (eight domains).

Components were coded using ICF numbers corresponding to second-level domains, third-level categories and fourth-level qualifiers. The ICF classification system categorises different aspects of functioning and disability into various domains and qualifiers. These domains include body functions, body structures, activities, participation and environmental factors. The qualifiers used in the ICF include level qualifiers, performance qualifiers and contextual factors.

The *Rehab4all* application helped reduce human coding errors. When instances arose where direct coding of the reported functional issue was not feasible, essential concepts from the assessment tool or outcome measures employed for appraising functionality and vulnerability were employed to deduce activity limitation or impairment, following ICF guidelines (De Moura et al. 2019).

### Ethical considerations

This article does not contain any studies involving human participants performed by any of the authors.

## Implications and recommendations

After conducting searches across multiple databases, a total of 3665 articles were identified. Subsequent removal of duplicate and screening of titles and abstracts resulted in 73 potentially relevant articles. After full text retrieval, 21 additional articles were excluded. A further 13 articles were excluded because of a lack of prevalence information on functioning problems. Finally, 39 studies met the criteria for inclusion in our review.

**FIGURE 1:**
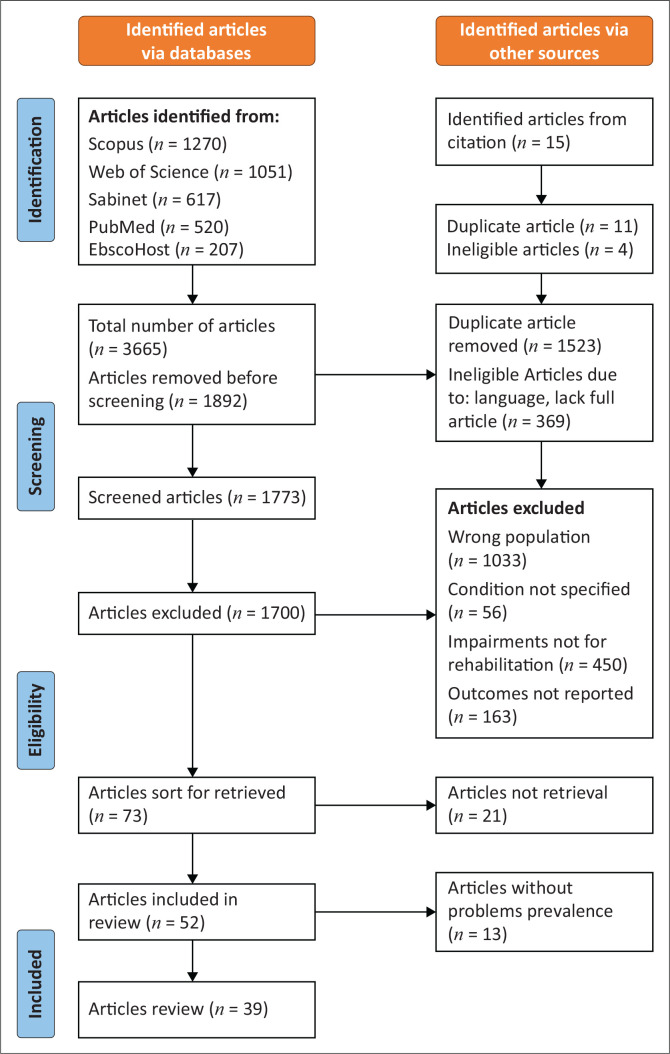
PRISMA flow diagram.

The PRISMA flow diagram (Figure 1) comprehensively outlines the selection process for articles at each phase, including specific reasons for exclusion.

### Study characteristics

Sample size totalled to 15620 study participants. The mean age for the sample could not be calculated as some studies did not report age as a mean; instead, they reported either the median or ranges within specific age groups. The studies were distributed across various settings: 26% (*n* = 10) were conducted at the community settings, 26% (*n* = 10) in primary healthcare units, 26% (*n* = 10) in specialised hospitals, and 7% (*n* = 3) at the hospital level. Regarding geographical location, 69% (*n* = 27) of the studies were conducted in urban area, 23% (*n* = 9) in rural settings, while 8% (*n* = 3) did not report on geographical location. A total of 74% (*n* = 29) of the studies were cross-sectional, 13% (*n* = 5) surveys, 3% (*n* = 1) cohort studies, while quasi-experimental studies constituted the remaining 2% (*n* = 27).

### Conditions associated with functioning problems within the global burden of disease in Kenya

The scoping review identifies the following top five conditions: major depressive disorder, HIV, low back pain, fractures and osteoarthritis as the main conditions significantly contributing to functioning problems in Kenya, as depicted in Table 1. The study considered conditions contributing the most to YLDs, that were reported in three articles and beyond because of the limited number of articles available for review.

**TABLE 1:** Conditions associated with functioning problems.

Condition	Sub-category	Number of articles
Major depressive disorder	-	7
HIV only	-	6
Low back pain	Mechanical	4
	Radiculopathy	1
Fracture or dislocation	Lower limb	3
	Spinal fracture	2
Osteoarthritis	Osteopathies	2
	Others	2
	Multiple sites	1
DM type II	-	2
Blindness	-	2
Schizophrenia	-	2
Digestive congenital anomalies	-	1
Congenital musculoskeletal	-	1
Stroke, unspecified	-	1
Chronic obstructive pulmonary disease, unspecified	-	1
Hypertensive heart disease	-	1

DM, diabetes mellitus; HIV, human immunodeficiency virus.

### Functioning problems

The *Rehab4all* application identified 19 different functioning problems through a literature review, irrespective of the prevalence or the number of articles discussing them. These documented problems included mobility, such as walking difficulties (unspecified, long distance), sensory problems including numbness/paraesthesia, as well as various types of pain (back pain, unspecified pain, joint pain) among others (Table 2).

**TABLE 2:** Functioning problems and the corresponding number of articles.

Problem	Number of articles
Walking difficulties, unspecified	15
Depressed	11
Housework difficulties	7
Walking difficulties, long distances	6
Numbness or paresthesia	5
Pain, back	4
Pain, unspecified	3
Dependence with mobility	3
Pain, joints	2
Anxious, nervous, worried	2
Cognitive deficit	1
Social participation	1
Restricted motion	1
Return or ability to work problems	1
Insomnia or sleep disturbances	1
Stressed or distressed	1
Pain, lower limbs	1
Numbness (feet or toes)	1
Blind	1

### Conditions and their associated functioning problems

**FIGURE 2:**
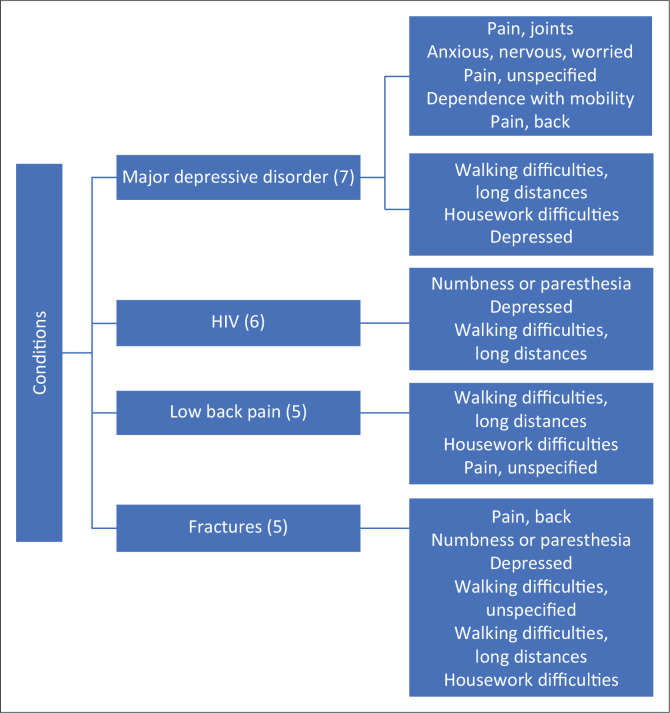
Common health conditions and associated functioning problems.

The scoping review articles identified the following top four conditions: (1) major depressive disorder (*n* = 7), HIV (*n* = 6) and low back pain and fractures (*n* = 5 for both). Figure 2 illustrates these conditions along with their corresponding functioning problems.

### Prevalence of functioning problems

**FIGURE 3:**
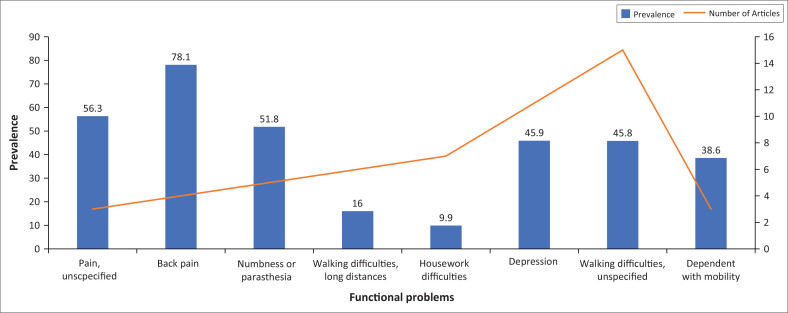
Prevalence of 5 top main functioning problems.

Scoping review identified eight functioning problems characterised by nature and/or body area, mainly associated with major depressive disorder, HIV, low back pain and fractures. The reported prevalence of these functioning problems exhibited a range spanning from 9.9% to 78.1%. In Figure 3, an overview of the prevalence is presented, focusing on functioning problems where prevalence data from at least three articles were available. Three articles and beyond were considered because of the limited number of studies that were available for review.

The most prevalent problems were back pain (78.1%), pain unspecified (56.3%), and significant prevalence was also noticed in numbness/paraesthesia (51.8%).

### Breakdown of the functioning problems into international classification of functioning, disability and health domains

#### Sensory and pain problems

Sensory and pain problems were identified. The highest prevalence of 78.1% was reported for back pain in five articles, and the prevalence for problems associated with numbness and paraesthesia was reported at 51.8% in four articles.

#### Mobility problems

The most prevalent mobility problems were walking difficulties (45.8%) reported by 15 articles while walking difficulties and long distances (16%) were reported by 6 articles.

### Mapping to the international classification of functioning, disability and health framework

Functioning problems were mapped by the *Rehab4All* tool to the ICF domains and categories using the outcome measures used in the studies (Table 3). Different articles used different outcome measures to assess functioning problems; for instance, Cresswell et al. (2020) used World Health Organization Disability Assessment Schedule (WHODAS-12), for functional assessment, while Kwobah et al. (2021) used Generalised Anxiety Disorder 7 (GAD 7) for assessment of mental disorders. The identified functioning problems covered four of the eight body function domains, one of the eight body structure domains and four of the nine activity limitation and participation domains (Charumbira et al. 2022b).

**TABLE 3:** Functioning problems mapped into International Classification of Functioning, disability and health and related outcome measures.

Problem	ICF Domain	ICF Code or Category	Outcome measure
Walking difficulties, unspecified	d4 Mobility	D450 Walking	WHODAS-12 [64, 73, 90] SF-36 [78], HARRIS SCORE [79], T-score [81]
	b2 Sensory functions and Pain	280 Pain sensation	Visual Analogue Scale (VAS), Pain Rating Scale (PRS) [75]
**Social participation**			
Housework difficulties	d6 Domestic life	d640 Doing house work	WHODAS-12[1], IEFF-5 [68], SF-36 [69], Family Reported Outcome Measure (FROM-16) [75]
Depression	b1 Mental function	b152 Emotional function	Centre for Epidemiologic Studies–Depression (CES-D) [66], SF-8 [66], Beck Depression Inventory (BDI- II) [59], Beck Anxiety Inventory (BAI) [73] Generalised Anxiety Disorder 7 (GAD 7) [77], Patient Health Questionnaire (PHQ 9) [77]
	b2 Sensory functions and Pain	280 Pain sensation	Visual Analogue Scale (VAS) [75]
Numbness or paresthesia	b2 Sensory function and pain	b270 Sensory related to temperature and other stimuli	Patient-Reported Peripheral Neuropathy [85, 91]
Numbness, feet or toes	b2 Sensory function and pain	b270 Sensory related to temperature and other stimuli	Treatment-induced Neuropathy Assessment Scale (TNAS)
Pain, unspecified	d7 Interpersonal interaction and relationships	d710 Basic interpersonal interaction	Visual Analogue Scale (VAS) Pain Rating Scale (PRS)
Stress or distressed	b1 Mental function	b152 Emotional function	Generalised Anxiety Disorder 7 (GAD 7) [77], Patient Health Questionnaire (PHQ 9) [77], Event Scale-Revised (IES-R), [89] Impact of Event Scale (IES)
Walking difficulties, long distance	d4 Mobility	d455 Moving around	WHODAS-12 [64, 73, 90] SF-36 [78], HARRIS SCORE [79], T-score [81]
Cognitive deficit	b1 Mental function	b117 Intellectual function	Severe Impairment Battery [82], Mini Mental State Examination and Alzheimer Disease [93] Assessment Scale, cognitive subscale, The Digit Span Forward Digit Span Backward and Trail Making Test-A
Pain, joints	b7 Neuromusculoskeletal and movement	7b80 Sensation related to muscle and movement functions	Visual Analogue Scale (VAS) Pain Rating scale (PRS)
	b2 Sensory functions and Pain	280 Pain sensation	Visual Analogue Scale (VAS), [75] Pain Rating scale (PRS) [92]
Restricted motion	b7 Neuromusculoskeletal and movement	b710 Mobility of joint functions	Osteoarthritis index (WOMAC) [86]
Anxious, nervous, worried	b1 Mental function	b152 Emotional function	Generalised Anxiety Disorder 7 (GAD 7) [82] Hospital Anxiety and Depression Scale, The Trait Anxiety Scale (T-Anxiety) The Beck Anxiety Inventory, the anxiety subscale of the Hospital Anxiety, Depression Scale
Insomnia or Sleep disturbances	b1 Mental function	b134 Sleep function	Pittsburgh Sleep Quality Index (PSQI) [16]
Pain, lower limb	b7 Neuromusculoskeletal and movement	b780 Sensation related to muscle and movement	Visual Analogue Scale (VAS) Pain Rating scale (PRS), [75]
Dependence with mobility	d4mobility	d455 Moving around	Wheelchair Skills Test Questionnaire [21] Wheelchair Use Confidence Scale for Manual Wheelchair [21] Efficiency of Assistive Technology and Services 6D Forms [21]
Pain, back	b7 Neuromusculoskeletal and movement	b780 Sensation related to muscle and movement	Visual Analogue Scale (VAS) [75] Neuropathic symptoms and Signs (S-LANSS) [88, 90] Oswestry Disability Index(ODI) [88]

ICF, International Classification of Functioning, disability and health; WHODAS, World Health Organization Disability Assessment Schedule.

### Mapping of functioning problems

**FIGURE 4:**
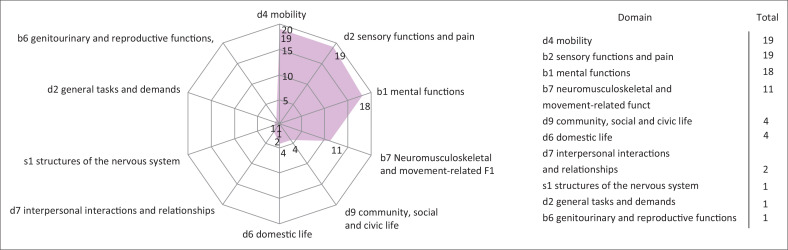
Mapping of functioning problems.

Among all the functioning problems (*n* = 80) identified, the majority involved mobility (*n* = 19), sensory functions and pain 24% (*n* = 19) and mental 24% (*n* = 18). Likewise, domestic life was also affected 5% (*n* = 4) and general tasks and demand 1% (*n* = 1) (Figure 4). Certain functioning problems extended across multiple ICF domains; for instance, housework difficulties spanned across four domains, while dependencies in mobility and joint pain each spanned across two domains. Most studies provided ample information, enabling the coding of identified functioning problems up to the fourth level. Fourth-level qualifiers were used to specify the exact degree of impairment in functioning, from complete absence of difficulty to a full extent of disability. To illustrate, for added specificity on mobility problems, the activity of lifting and carrying objects was further categorised, such as d4300 lifting.

## Discussion

The aim of the current scoping review was to identify and summarise the existing body of evidence from peer-reviewed literature with regard to functioning problems associated with health conditions with greatest disease burden in Kenya. When mapped to the ICF, the most prevalent functioning problems were related to mobility, sensory and pain, and mental health.

The articles included in the review presented environments in both rural and urban settings, offering valuable insights into how functioning problems are influenced by the environment. This highlights the widespread nature of these problems and reinforces the evidence presented by Braveman and Gottlieb (2014) on the role environmental factors play in health outcomes. Similar to the findings of Natarajan et al. (2023), poorly planned or maintained infrastructure such as a lack of ramps, uneven sidewalks or limited public transportation can worsen mobility challenges. These findings should be considered when developing interventions and policies aimed at addressing the diverse barriers individuals face in different environments.

The articles included in the review had a notable focus on densely populated areas and accessible tertiary health facilities. This suggests potential disparities in the healthcare research infrastructure, highlighting limited resources particularly at the primary healthcare level in Kenya, which supports MacLellan, Turnbull and Pope (2022), in a study on infrastructural research barriers. The lack of health research infrastructure including technological resources, human resources and funding mechanisms may negatively impact the conduct of high-quality research in primary healthcare settings (Zakaria, Grant & Luff 2021). The deficiency in our study causes skewed representation of health data, limiting the applicability of research findings to the entire populations. It also leads to a disproportionate reliance on external sources for health data and interventions. This dependency can contribute to a lack of autonomy in addressing local health challenges and tailoring interventions to the unique socio-cultural contexts. However, the results have the potential to inform policy decisions, funding allocation strategies and capacity-building initiatives aimed at reducing disparities in health research infrastructure.

Our main finding aligns with Charumbira et al. (2022a), where mobility, pain and mental health-related problems were identified as the most prevalent functioning problems. Likewise, Matter and Eide (2018) reported walking difficulties and stair climbing problems in Botswana and Swaziland, respectively. Identifying specific functioning problems in Kenya may enable policymakers and healthcare providers to prioritise resources for interventions. This may lead to funding allocation and programmes improving access to mobility aids, pain management and mental health support. Mobility issues and pain can hinder individuals’ work and daily activities, impacting productivity. Addressing these problems could enhance economic participation and yield broader socioeconomic benefits. Recognising mental health prevalence highlights the need to support social support systems, expand services, and provide healthcare professional training. Our findings can also drive research and innovation for tailored interventions as well as policy development aimed at enhancing health outcomes and quality of life for Kenyan citizens, integrating strategies into broader healthcare initiatives.

This review identified 19 distinct functioning problems requiring rehabilitation. However, rehabilitation faces challenges in providing services across the various health system levels and in sectors beyond healthcare. Neill et al. (2023), in a study on prioritising rehabilitation in LMICs, found that rehabilitation is affected by fragmented governance across various sectors and stakeholders, as well as weak health systems. This negatively affects patient engagement, leading to suboptimal rehabilitation care and lower levels of care satisfaction (Maphumulo & Bhengu 2019). Literature has highlighted the need for a novel approach to effectively manage functioning problems associated with health conditions (Coulter & Oldham 2016). Ekman and Swedberg (2022) propose a person-oriented care model to comprehensively address the diverse range of functioning problems.

The majority of functioning problems identified in the present review mapped within the ICF framework demonstrated interconnectedness and spiralling, where a single condition can manifest various functioning problems spanning across different domains and categories. As a result, targeting a particular functional problem has the capacity to concurrently mitigate the corresponding impact on other affected domains. For example, addressing reduced range of motion in the lower limbs can enhance walking ability, which may in turn alleviate challenges related to domestic life. This interconnectedness reflects the holistic nature of rehabilitation interventions, as discussed by Jasemi et al. (2017). Therefore, challenges or improvements in one aspect of functioning may set off a chain reaction, either exacerbating or alleviating difficulties in other domains. This aligns with the ICF’s biopsychosocial model, where body functions, activities and participation are dynamically interconnected. As suggested by Cadel et al. (2022), adopting a comprehensive approach to rehabilitation, addressing both specific impairments and broader life challenges, can significantly enhance overall function and quality of life. Stucki et al. (2018) suggests that rehabilitation is becoming a key health strategy in the 21st century, although it may fall short if conducted in isolation. The effectiveness of rehabilitation is enhanced when it is part of a multidisciplinary approach that addresses the complex, multifaceted nature of functioning problems related to different health conditions (Bøgdal et al. 2021; Builova & Marchenkova 2020; WHO 2021). Isolated rehabilitation may lack the broader perspective needed to address underlying issues contributing to functioning problems, such as psychosocial factors, environmental considerations and individualised patient needs.

The limited number of identified problems in our study can serve as a snapshot, providing valuable insights to raise awareness among rehabilitation professionals regarding the contextual presentation of functioning problems. This can assist clinicians in the recognition, assessment and development of targeted, patient-centred rehabilitation plan as suggested by Gonzalez–Suarez et al. (2015). Understanding functioning provides a clearer insight into the burden associated with a health condition and its impact on a person’s life roles (Seger, Grotkamp & Cibis 2017). This enhanced awareness contributes to a more comprehensive and informed approach to rehabilitation strategies, ultimately improving the overall effectiveness of patient care. Informed patients are empowered to participate in healthcare decisions, aligning treatments with their preferences, which leads to better engagement in rehabilitation and improved quality of life (Coulter & Oldham 2016; Van der Laag et al. 2021).

Integrating rehabilitation into a comprehensive healthcare framework improves functional outcomes by addressing the multifaceted nature of patient conditions (WHO 2021). Evidence shows that rehabilitation plays a critical role not only in restoring function but also in prevention, curative and palliative care (Conradie et al. 2022; Janerka, Leslie & Gill 2023; Lee & Lee 2021; Rauch et al. 2019). In Kenya, the integration of rehabilitation into primary healthcare could better address the underlying functioning problems in rural populations, where 64% of the population resides, yet access to rehabilitative services is extremely limited (Oyugi 2019). With 3.5% of the population living with a disability (Owino 2020), the scope for rehabilitation services is vast, especially given the underreporting of these figures (United Nations 2019). This evidence suggests that by embedding rehabilitation services into a broader system, Kenya can improve access and outcomes for those in resource-limited settings.

The Ministry of Health in Kenya has committed to improving rehabilitation services, but barriers remain, particularly in rural and primary healthcare settings (Owino 2020; Oyugi 2019). To enhance service quality and access, the development of rehabilitation professionals is crucial. Evidence supports that the effectiveness of rehabilitation depends on the competency of healthcare personnel, particularly in terms of tailored knowledge, skills and attitudes suited to resource-constrained settings (Heine, Derman & Hanekom 2022; WHO 2021). The introduction of Bachelor of Science programmes in rehabilitation fields is vital for building this workforce, ensuring professionals are equipped to meet Kenya’s rehabilitation needs, especially in primary healthcare (Ministry of Health 2023). Investments in education and skill development will help address the country’s rehabilitation gap and improve overall healthcare outcomes.

### Strengths

The research leveraged the ICF as a standardised framework. It provides a structured and internationally recognised approach for describing functioning problems among adults in low-resource settings and enhances the reliability and comparability of the findings.

The use of the web-based application *Rehab4all* facilitated a clear process for extracting data. It streamlined data collection, enhanced efficiency and allowed updates.

### Limitations

There is a scarcity of articles reporting on functioning problems within the GBD in Kenya. The available literature may not sufficiently cover the topic, impacting the depth of understanding of functioning problems in the context of the GBD. Because of the scarcity of articles, the generalisability and comprehensiveness of the research findings regarding functioning problems in the Kenyan population may be constrained. This limitation highlights a potential gap in knowledge and raises questions about the extent to which the findings can be applied broadly.

### Need for further research

The limitation identified in the scoping review emphasised the necessity for further research to address the lack of information. While acknowledging this limitation is important, it also serves as a call to action, indicating the importance of future studies to fill the gaps and provide a more understanding of the impact of functioning problems on the overall burden of disease in Kenya.

## Conclusion

This scoping review identifies prevalent functioning problems associated with health conditions leading to disability, mainly observed in primary healthcare and community settings in Kenya. When mapped to the ICF, the most prevalent functioning problems were related to mobility, sensory and pain, and mental health. This highlights the necessity for innovative strategies to improve rehabilitation services, especially in areas lacking professionals. County-level health departments can be reformed to provide health promotion and prevention services addressing these problems. Early detection through routine screening in primary healthcare and community settings is crucial, using accessible tools. Disseminating knowledge and implementing self-management strategies within communities, utilising the existing healthcare workers, can be effective. These adjustments can improve population health outcomes and support universal health coverage goals.

## Footnotes

1 Grey literature refers to research and materials that are not formally peer-reviewed but still contribute valuable insights to research and practice.
